# Tanshinon IIA Injection Accelerates Tissue Expansion by Reducing the Formation of the Fibrous Capsule

**DOI:** 10.1371/journal.pone.0105756

**Published:** 2014-08-26

**Authors:** Qingxiong Yu, Lingling Sheng, Mei Yang, Ming Zhu, Xiaolu Huang, Qingfeng Li

**Affiliations:** 1 Department of Plastic and Reconstructive Surgery, Shanghai Ninth People's Hospital, Shanghai Jiao Tong University, School of Medicine, Shanghai, P.R. China; 2 Division of Plastic Surgery, Southern Illinois University School of Medicine, Springfield, Illinois, United States of America; University of California, San Francisco, United States of America

## Abstract

The tissue expansion technique has been applied to obtain new skin tissue to repair large defects in clinical practice. The implantation of tissue expander could initiate a host response to foreign body (FBR), which leads to fibrotic encapsulation around the expander and prolongs the period of tissue expansion. Tanshinon IIA (Tan IIA) has been shown to have anti-inflammation and immunoregulation effect. The rat tissue expansion model was used in this study to observe whether Tan IIA injection systematically could inhibit the FBR to reduce fibrous capsule formation and accelerate the process of tissue expansion. Forty-eight rats were randomly divided into the Tan IIA group and control group with 24 rats in each group. The expansion was conducted twice a week to maintain a capsule pressure of 60 mmHg. The expansion volume and expanded area were measured. The expanded tissue in the two groups was harvested, and histological staining was performed; proinflammatory cytokines such as tumor necrosis factor-α (TNF-α), interleukin-6 (IL-6) and interleukin-1β (IL-1β) and transforming growth factor-β (TGF-β) were examined. The expansion volume and the expanded area in the Tan IIA group were greater than that of the control group. The thickness of the fibrous capsule in the Tan IIA group was reduced with no influence on the normal skin regeneration. Decreased infiltration of macrophages, lower level of TNF-α, IL-6, IL-1β and TGF-β, less proliferating myofibroblasts and enhanced neovascularization were observed in the Tan IIA group. Our findings indicated that the Tan IIA injection reduced the formation of the fibrous capsule and accelerated the process of tissue expansion by inhibiting the FBR.

## Introduction

The tissue expansion technique has been in use for many years in plastic surgery. It's a process of implanting a silicon sac subcutaneously and regularly injecting saline into the sac, in this process, new skin forms under the mechanical stretch, providing a supply of tissue similar in color, structure and adnexal distribution to the adjacent skin for a perfect repair [1]. Tissue expansion is a very useful technique and has a wide range of clinical applications, especially for repairing of large skin defect.

Tissue expansion is a safe and reliable method for reconstruction; however, it is also a time-consuming procedure. In clinical practice, 4–6 months are usually needed to achieve adequate expansion. Such long time expansion not only causes distress in patients, but also increases the incidence of complications such as infection, and the rupture or exposure of the tissue expander.

According to previous studies of tissue expansion, there are two important histological changes occurring during expansion. The first one is tissue regeneration, which is correlated with cell proliferation and neovascularization of expanded tissue. The other change is the formation of fibrous capsule. In our previous studies, we have been trying to promote tissue expansion by enhancing skin regeneration. Stem cells, such as bone marrow derived stem cells (BM-MSCs) [2], adipose tissue derived stem cells (ADSCs) [3], and stromal vascular fraction (SVF) [1] have been found effective in accelerating tissue expansion and promoting skin growth. In this study, we aimed to reduce fibrous capsule formation during tissue expansion, which may potentially increase the efficiency of tissue expansion technique and the efficacy of stem cell therapy during tissue expansion.

The fibrous capsule forms, because the tissue expander, as a foreign body, initiates a foreign body response (FBR) after implanted in vivo, which is characterized by macrophage infiltration and fibrotic encapsulation around the expander to “seal off” the expander [4]. The Jun N-terminal kinase (JNK) and NF-κB inflammatory pathways and proinflammatory cytokines such as tumor necrosis factor-α (TNF-α), interleukin-6 (IL-6) and interleukin-1β (IL-1β) are involved in the FBR, according to our previous experiment [5].

During tissue expansion, inelastic capsule augments the mechanical resistance to the expander, which significantly prolongs the expansion time [6]. The capsule also causes the contracture of expanded flap, which reduces available expanded skin for repair surgery, impairs reconstruction, and usually leads to extra surgeries or even re-expansion. Many efforts have been made to explore a wide range of substances, topical or systemic delivered, to reduce the formation of fibrous capsule to shorten the tissue expansion process [7], [8]. However, these agents are limited in clinical application because of their toxic effects.

**Figure 1:**
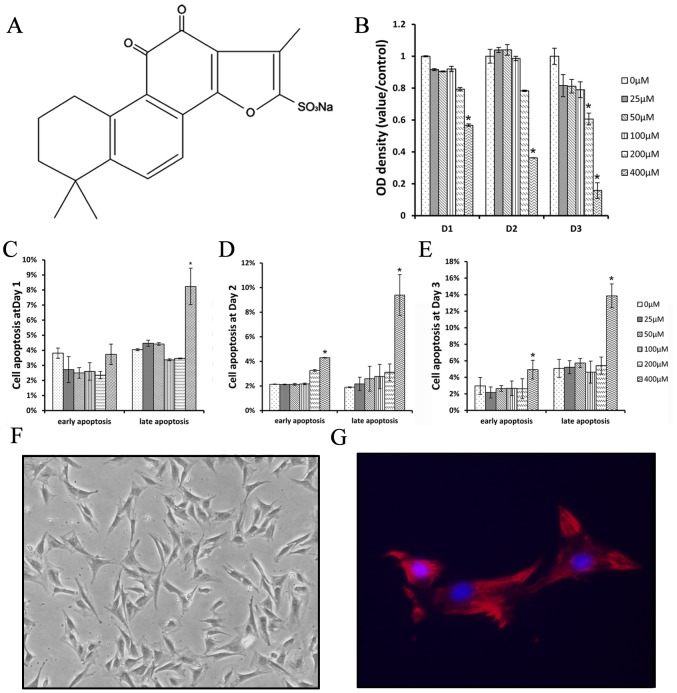
The molecular structure of Tan IIA used in the study (A). The effect of Tan IIA on the viability (B) and apoptosis (C–E) of dermal fibroblasts *in vitro* from days 1 to 3. Dermal fibroblasts demonstrate a spindle shape under optical microscope (F, 40×) and are positive stained with vimentin (G, 200×). There was no remarkable change as the concentration of Tan IIA varied when compared with 0 µM, except with 400 µM, showing that a low Tan IIA concentration could not influence the fibroblasts. Mean ± SD; *P<0.05.

Tanshinone (Danshen) is an herbal medicine derived from the dried root of *Salvia miltiorrhiza Bunge*, which has a wide clinical use in China and other countries for hundreds of years [9], [10]. Tanshinon IIA (Tan IIA), the main component of Tanshinone, has drawn extensive attention because of its therapeutic efficacy in cardiovascular diseases, metabolic diseases and cancers. As a multi-target drug, its molecular targets include transcription factors, ion channels, apoptosis regulating proteins, growth factors and inflammatory mediators [11]. The Tan IIA we used in this study is a finished pharmaceutical product purchased from Shanghai No. 1 Biochemical & Pharmaceutical Co. Ltd. (Shanghai, P.R. China), The English name of this product is Sulfotanshinone Sodium Injection, it is a sulfonated form of Tan IIA. The formula of this compound is C19H17NaO6S (Fig. 1A). The Tan IIA acquires enhanced water solubility in a sulfonated form and the drug efficacy is improved. This chemical drug meets a criterion of Chinese sanitation industry standard WS-10001-(HD-1014)-2002. The effective component accounted for more than 98% of the chemical and the impurity content is below 1%.The aim of this study was to evaluate the effectiveness of systemically delivered Tan IIA on the process of tissue expansion and the formation of the fibrous capsule during tissue expansion.

## Materials and Methods

### 1. Ethics Statement

The Animal Care and Use Committee of Shanghai Jiaotong Medical University approved all of the experiments in accordance with the Declaration of the National Institutes of Health Guide for Care and Use of Laboratory Animals (Publication No. 85-23, revised 1996). All of the surgeries were performed under sodium pentobarbital anesthesia, and all efforts were made to minimize suffering.

### 2. Animal preparation

Sprague-Dawley male adult rats, weighing 220–250 g each, were obtained from the Animal Center (Shanghai Experimental Animal Center, China). The rats were kept in a temperature-controlled habitat under a 12 h light/dark cycle and fed a standard laboratory diet and watered libitum.

### 3. Cell culture and Tan IIA treatment

Three-day-old SD rats were sacrificed with 3% sodium pentobarbital to harvest the skin fibroblasts according to the method reported previously [12]. The cells of passage 3–4 were seeded at 1×10^3^ cells per well in 96-well plates with 100 µl culture medium. After being treated with Tan IIA (Shanghai No. 1 Biochemical & Pharmaceutical Co. Ltd, Shanghai, China) of different concentrations, 10 µl of Cell Counting Kit-8 (CCK-8) solution (Dojindo, Kumamoto, Japan) was added to each well, and the plates were incubated at 37°C for 3 h. The supernatants were aspirated and transferred into a new 96-well plate, and the optical density at 450 nm was read on a Microplate Reader (Bio-Rad, Hercules, CA, USA). The effect of Tan IIA on the viability of the adherent monolayer fibroblasts was presented as the % of cytoviability using the following formula: % cytoviability  =  A450 of treated cells/A450 of control cells ×100%. Three independent experiments were performed.

The cell apoptosis was measured with Annexin V-FITC and PI (BD Bioscience, San Jose, CA, USA) using a detection kit. The cells were harvested, washed once with cold PBS, and resuspended in 500 µl of binding buffer with a number of 1×10^5^ cells, followed by staining with Annexin V-FITC and PI solution in the dark for 15 min at room temperature. The cells were centrifuged at 1000 rpm for 5 min, and the pellets were resuspended in 500 µl of binding buffer. After filtration, the percentage of cell apoptosis was determined using flow cytometry (BD FACSCalibar, San Jose, CA, USA).

### 4. Tissue expanding model and Tan IIA injection

Forty-eight SD rats were anaesthetized with 3% sodium pentobarbital at 0.13 ml/100 g and shaved. The 10 ml tissue expanders were implanted in the back of the rats as previously described, and 10 ml of physiological saline was injected through the tissue expander pot immediately after the implantation [7]. The expansion was conducted twice a week with an internal pressure maintained 60 mmHg, monitored by a simple self-made apparatus composed of a sphygmomanometer and a three-way pipe [1]. After a preliminary study, we chose 10 mg/kg/day as an appropriate dose for Tan IIA injection; The rats in the Tan II A group received daily intraperitoneal injection of Tan IIA for 28 days, and the rats in the control group received physiological saline injection.

### 5. Expansion volume and expanded area

The expansion ended after 4 weeks, and the water volume in the expander pockets of each group was added to perform the statistical analysis. The expanded skin was scanned using a Konica Minolta Vivid 910 fw 3D laser scanner (Konica Minolta, Tokyo, Japan) and the images were imported into Mimics CAD/CAM software (Konica Minolta, Tokyo, Japan) to create three-dimensional images of the expanded tissue to measure area of the expanded skin.

### 6. Histological staining

Expanded tissue specimens were collected at day 7, 14 and 28 (n = 6 for each group) postoperatively, and fixed with 4% paraformaldehyde for 24 h, and embedded in paraffin. Histological staining was performed on 4 µm section. The hematoxylin-eosin (H&E) staining was performed to observe the thickness of the whole skin layers and the fibrous capsule. Immunohistochemistry staining with vimentin (Santa Cruz, CA, USA) for fibroblasts, with CD68 (Abcam, Cambridge, UK) for macrophages, with α-smooth muscle actin (α-SMA; Santa Cruz, CA, USA) for myofibroblasts, with CD31 (Abcam) for capillaries and proliferating-cell nuclear antigen (PCNA; Abcam) for proliferating cells was performed. The steps were identical to those same as described in a previous report [3].

### 7. Protein level of cytokines

For the detection of growth factors, 0.5 g of tissue on the above area of tissue expander from each group was collected (n = 6 for each group). The tissues were homogenized in 500 µl of tissue protein extraction reagent (CWBIO, Beijing, China) and 5 µl of PMSF (Sigma-Aldrich). After centrifuging at 10000 rpm for 10 min, the supernatant was collected for the assay of TNF-α, IL-1β, IL-6 and TGF-β using a Protein Quantibody Array kit according to the manufacturer's instructions (R&D Systems, Minneapolis, USA). The signals were visualized by using a laser scanner equipped with a Cy3 wavelength. The data were extracted with microarray analysis software programs (GenePix, ScanArray Express, ArrayVision, or MicroVigene).

### 8. Statistical analysis

All of the quantitative variables were analyzed with the SPSS 13.0 program (SPSS, Inc., Chicago, IL, USA). All of the values were expressed as mean ± SD. The analysis of variance followed by Student's test was used to determine the significant differences between the two groups. Values of *P*<0.05 were considered statistically significant.

## Results

### 1. Effect of Tan IIA on the viability and apoptosis of fibroblasts

The effect of Tan IIA on the growth of the fibroblasts was examined with CCK-8 and Annexin V/PI. After exposure to Tan IIA at concentrations of 0, 25, 50, 100, 200, 400 µM, the viability and apoptosis of the fibroblasts showed no evident change when compared with that without Tan IIA treatment, except at the concentration of 400 µM. In this high concentration, the viability of fibroblasts was inhibited and the percentage of apoptosis was increased. All of these results suggested that a low concentration of Tan IIA should not influence the growth of fibroblasts (Fig. 1 B–E).

### 2. Tan IIA accelerated tissue expansion

**Figure 2:**
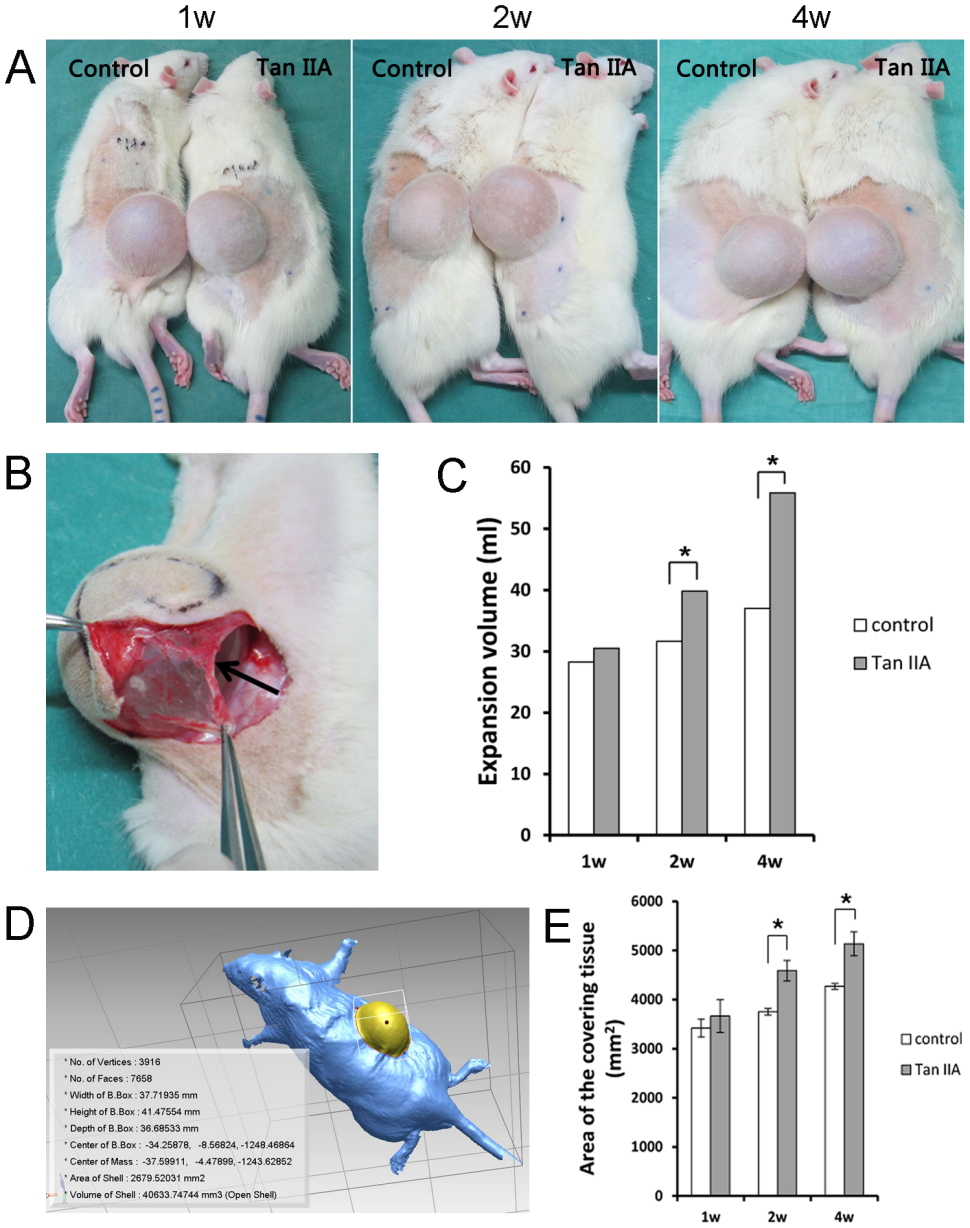
After the Tan IIA injection, the expansion volume was much greater than that of the control group at day 14 and 28 (A and C). The arrow in B showed the fibrous capsule formed around the tissue expander. The expanded area was measured using the Konica Minolta Vivid 910 fw 3D laser scanner (D), and there were significant differences between the two groups at day 14 and 28 (E). Mean ± SD; *P<0.05.

The inflation volume was measured to evaluate the effect of Tan IIA on tissue expansion. Four weeks later, the Tan IIA group had a higher inflation volume (55.88±5.56 ml) when compared with the control group (37.00±0.82 ml) (n = 6, *P*<0.01). The difference between the two groups appeared at the second week (39.83±1.57 ml in the Tan IIA group and 31.63±1.25 ml in the control group (n = 6, *P*<0.05)). Consistent with the expansion volume, the expanded area began to show a difference at day 14 and became evidently at day 28. There was no significant difference at day 7 (n = 6, *P*>0.05) (Fig. 2).

### 3. The thickness of the expanded skin and the fibrous capsule

**Figure 3:**
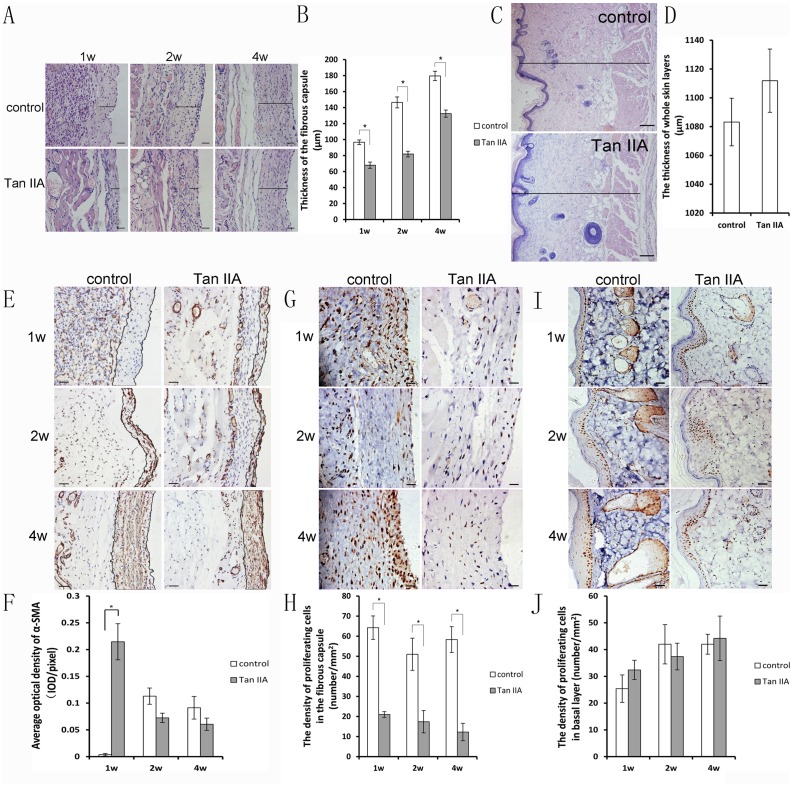
The thickness of the fibrous capsule in the Tan IIA group was much less than that in the control group at all of the time points (A and B), and the thickness of the whole skin layers, except the fibrous capsule, showed no difference (C and D). There were fewer α-SMA positive myofibroblasts in the fibrous capsule in the Tan IIA group (E and F), and their proliferation was much lower (G and H). The proliferating cells in the basal layer in the two groups showed no difference (I and J). The area within the black lines in E was calculated for the α-SMA density. The scale bars in E, G and I were ×200. Mean ± SD; *P<0.05; Bar in C, 40 µm; Bar in E, G, and I, 200 µm.

The thickness of the entire skin layer was measured, including the epidermis, dermis and subcutaneous tissue. At day 28, the skin thickness in the Tan IIA group (1111.9±21.96 µm) showed no significant difference (n = 6, *P*>0.05) compared with the control group (1083.2±16.53 µm). The thickness of the fibrous capsule in the control group was much thicker than that in the Tan IIA group at all of the time points (n = 6, *P*<0.05) The fibrous capsule measured in this study was the internal layer in contact with the implant, which was irregular and filled with collagen. The layer of parallel fibers combined with the capillaries as well as the layer of panniculus carnosus was not included (Fig. 3).

### 4. The proliferating cells in the expanded tissue

The PCNA-positive cells were distributed in all of the layers in the expanded tissue, especially in the basal layer and at the tissue-material interface. There was no difference in the positive cells in the basal layer between the Tan IIA group and the control group at all of the time points (*P*>0.05), showing that Tan IIA did not influence skin growth. The density of the PCNA-positive cells at the tissue-material interface in the Tan IIA group was much less than that in the control group, which might contribute to the reduced formation of the fibrous capsule (Fig. 3).

### 5. Tan IIA injection enhanced the neovascularization of the expanded skin

**Figure 4:**
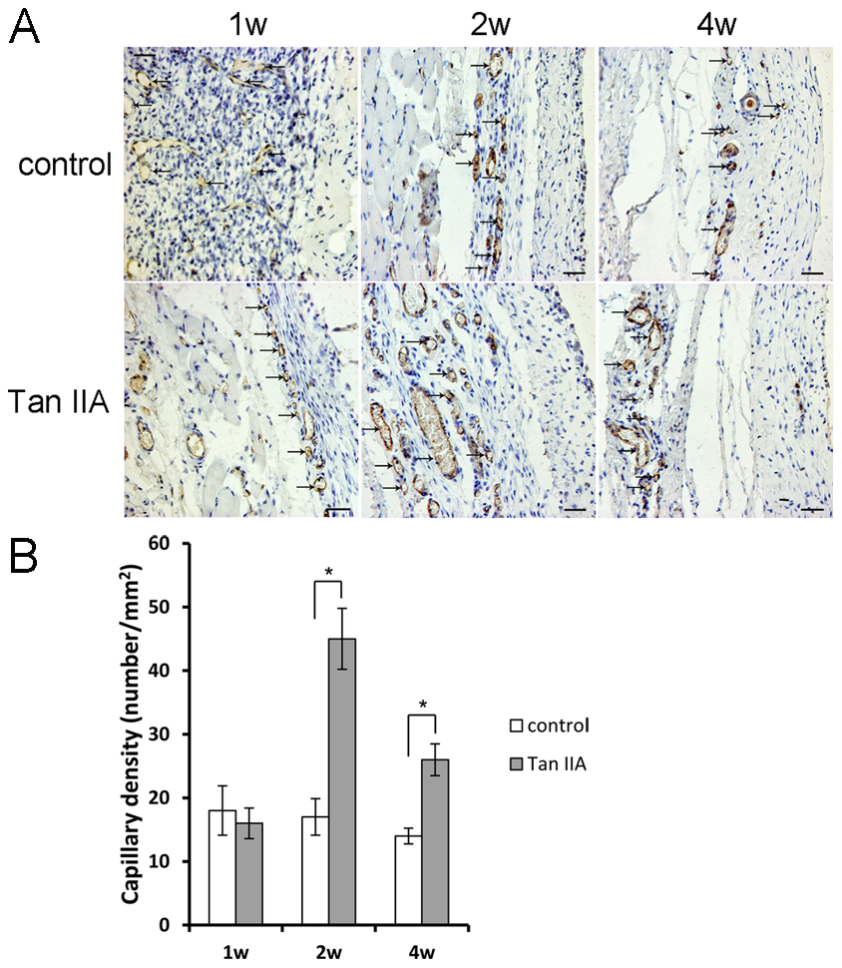
The capillary density in the membrana carnosa, which provides the blood supply for the fibrous capsule, was much higher in the Tan IIA group than in the control group at days 14 and 28, indicating that the Tan IIA injection could promote neovascularization. Mean ± SD; *P<0.05; Bar, 200 µm.

Anti-CD31 immunohistochemical staining was used to determine the degree of neovascularization of the membrana carnosa, which provide the blood supply for the fibrous capsule. No difference of capillary density was found between the two groups at day 7 (n = 10, *P*>0.05). However, the capillary density in Tan IIA group was clearly increased after 14 days (45±4.7/mm^2^ at day 14 and 26±2.50/mm^2^ at day 28), compared with the control group (17±2.78/mm^2^ at day 14 and 14±1.24/mm^2^ at day 28) (n = 10, *P*<0.05), signifying that systematically injection of Tan IIA could effectively enhance the viability of the capillary vessel (Fig. 4).

### 6. Tan IIA inhibited the infiltration of macrophages and secretion of inflammatory cytokines

**Figure 5:**
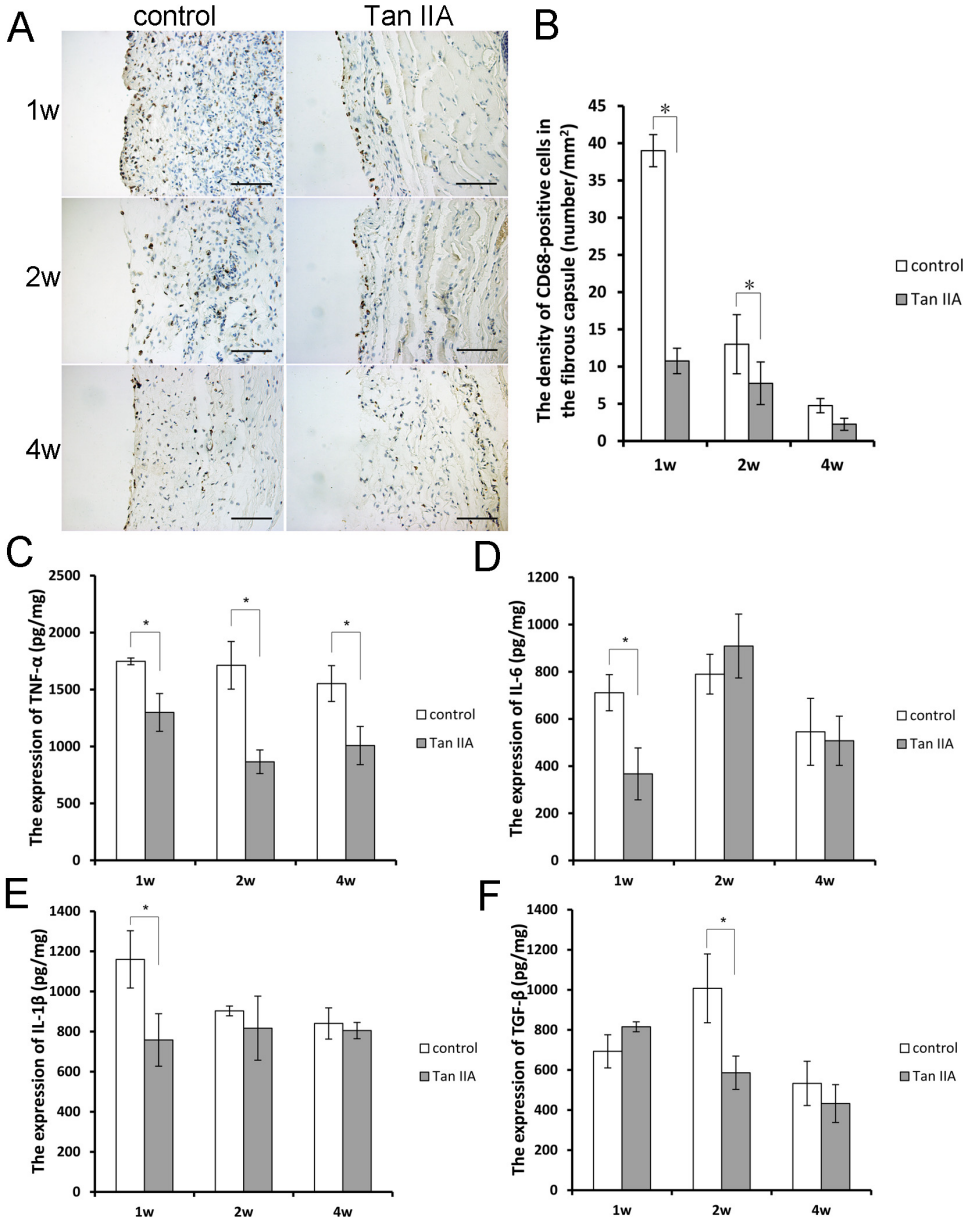
There was less infiltration of the CD68-positive macrophages at the tissue-material interface in the Tan IIA group when compared with the control group, particularly at day 7 (A and B). The inflammatory cytokines, IL-6, TNF-α, IL-1β and TGF-β showed higher expression in the expanded tissue at all of the time points in the control group, and after the Tan IIA treatment, these three cytokines presented lower expression (C–F). Mean ± SD; *P<0.05; Bar, 200 µm.

The macrophages showed a persistent infiltration, and were primarily located at the tissue-material interface in both the Tan IIA and control groups. However, less macrophages were observed in the tissue from the Tan IIA group compared with the control, especially at day 7 (Fig. 1). Inflammatory cytokines, IL-6, TNF-α, IL-1β and TGF-β showed higher expression in the expanded tissue at all of the time points in the control group, however, in the Tan IIA group, these four cytokines presented a lower expression (Fig. 5).

## Discussion

Repairing a defect resulting from wound or surgical resection is challenging, and the best tissue for such coverage is the one similar to the original. A local flap may be the best solution to this problem. However for a large defect, local flap cannot provide sufficient coverage. The use of tissue expanders presents a promising alternative. The advantages of tissue expansion were summarized by Schmidt et al. as follows: the best tissue match with defect areas, the lack of a wound at the donor site and the maintenance of the vascular supply [13]. However, tissue expansion is a time consuming procedure. During months of expansion, it is suffering and inconvenient for the patient and the probability of all kinds of unexpected events, such as infection and rupture, also increases. Finding a simple and effective measure to speed up the expansion process has very important clinical implication.

Capsule formation is a chronic-proliferative inflammation process [14], [15], and is determined by the physico-chemical properties of the implants as well as the various biological components, to which the implant is exposed after implantation [16]. Periprosthetic fibrosis is a reaction to a foreign body and begins at the moment of implantation of the prosthesis and lasts until its stabilization. Myofibroblasts are the main cells in the fibrous capsule. In the FBR process, dermal fibroblasts are activated and migrate to the fibrous capsule layer. Vimentin positive fibroblasts in the capsule tissue decrease over time, and some of them transdifferentiate into myofibroblasts under TGF-β regulation [17]. The activated myofibroblasts proliferate and synthesize collagen, resulting in material encapsulation [18], [19]. Our results showed that lower concentration of Tan IIA had no influence on the viability and apoptosis of fibroblasts, however, α-SMA positive myoblasts in the fibrous capsule was much less after Tan IIA application. It seems that Tan IIA could inhibit the trasndifferentiating of fibroblasts into myofibroblasts. This may relate to the inhibition of TGF-β expression. The reduced density of the proliferating myofibroblasts in the fibrous capsule led to less collagen deposition and fibrous capsule formation. The result was consistent with the study reported by Laitung et al [20], who found that less number of myofibroblasts in the tissue around tissue expanding devices led to lower capsular formation and contracture.

The cell proliferation in the stratum basele, which plays an important role in skin growth, was not influenced by the Tan IIA treatment. Similarly in the *in vitro* study, Tan IIA treatment with a low dosage did not inhibit the fibroblast proliferation and did not promote fibroblast apoptosis. The thickness of the whole skin layers was not influenced, and there was no difference between the two groups at the end of tissue expansion. These results indicated that the capsule formation in the Tan IIA group was inhibited but that the skin growth was not influenced. The greater inflation volume with the larger expansion area in the Tan IIA group was positively correlated with decreased capsule thickness. In our earlier experiment, 20 mg/kg of Tan IIA was injected every day, and at approximately 14 days, the expanded skin of some rats underwent necrosis. The result was consistent with our in *vitro* study, which showed that high concentration (400 µM) may inhibit the fibroblast proliferation and promote fibroblast apoptosis. So in this study we chose 10 mg/kg/day as a safe and effective drug dose.

The expanded tissue had a high vascularity that was considered superior to surgical delayed flaps [21]. High neovascularization of a tissue flap before transplantation is critical to its postoperative survival. The histologic examination showed that Tan IIA treatment could enhance angiogenesis, which was evidenced by the increased number of blood vessels, providing the newly formed tissue with sufficient nutriments and oxygen to improve the viability of the expanded skin. In this study, thickness of the fibrous capsule decreased, however, the capillary density was not decreased.

Fibrous capsule formation is a complicated multifactorial process. The severity of capsule contracture has a positive linear correlation with the degree of local inflammatory reactions [22]. When the tissue expanders are implanted, the injury and ischemia caused by the surgical procedure initiate an acute inflammatory cascade. In the long term expansion period, the acute inflammatory transfer into a chronic pattern because the tissue expander as a non-removable injurious stimuli. In this process a protein layer adsorbs to the biomaterial surface, and macrophages, the main cells participating into FBR, adhere to the surface via interaction of adhesion receptors with the adsorbed proteins [23]. Our results showed that Tan IIA markedly reduced the infiltration of CD68-positive macrophages and the expression of inflammatory cytokines in the local tissue. This chronic inflammatory inhibiting effect explained less fibrous capsule formation in the Tan IIA group. Tan IIA inhibits the chronic inflammatory which shows little relation with infection defense but a pathological state of the body to non-removable injurious stimuli. Chronic inflammation can always lead to tissue destruction and fibrosis, including the fibrous capsule formed after material implantation. So Tan IIA could inhibit fibrous capsule formation without infection defense function impairment. Since the use of Tan IIA has a long history and the long-term practice proved it to be a safe and effective traditional Chinese medicine (TCM) preparations, we think it is safe to use Tan IIA to reduce the formation of fibrous capsule around the expander. And in our experiment all rats receiving Tan IIA injection had no sign of infection.

Macrophages produce growth factors and fibrocyte-stimulating cytokines, such as IL-6, TNF-α, IL-1β and TGF-β [13]. In our study, the expression of these cytokines decreased in the Tan IIA group. The TNF-α secreted by macrophages could recruit more macrophages to the target tissue, and there is positive feedback between them. In our previous study, we demonstrated that the NF-κB and JNK pathways were the major pathways activated in the tissue expansion model [5]. NF-κB is one of the central players in the cascade, and it is initiated and plays a crucial role in regulating inflammatory signal transduction and cytokine production. Research showed that Tan IIA could suppress the TNF-α expression after cellular oxygen-glucose deprivation/recovery (OGD/R) in a dose-dependent manner and this suppression was mimicked by BAY 11-7082, a commercial NF-κB inhibitor [24]. Another study demonstrated that Tan IIA could reduce the levels of IL-6, TNF-α and IL-1β by markedly inhibiting the activation of NF-κB and the mitogen-activated protein kinases (MAPKs) pathways in a spinal cord injury model [25]. All these results indicated that Tan IIA could inhibit NF-κB pathway and down regulate the expression of kinds of inflammatory cytokines. IL-1 and TGF-β have been showed to have an important regulatory effect on fibroblast proliferation and collagen synthesis [26]–[28]. High levels of TGF-β and IL-1 could potentially result in significant fibrosis surrounding silicone implants *in vivo*. The TGF-β1 inhibitor peptide was significantly effective in achieving a reduction in periprosthetic fibrosis after silicone implants placements [29]. This explains the fibrous capsule formation inhibiting effect of Tan IIA in our experiment.

Our results suggested that pharmacological control of capsular formation and neovascularization in the course of tissue expansion was feasible by means of systemic application of Tan IIA. With Tan IIA, the expansion can proceed in a safer, more rapid and efficacious manner, relieving patients' discomfort and lowering the total cost of the procedure. Extensive study is still needed to test the long-term safety of Tan IIA and to evaluate its mechanisms of action.
